# *Cyclin A1* shows age-related expression in benign tonsils, HPV16-dependent overexpression in HNSCC and predicts lower recurrence rate in HNSCC independently of HPV16

**DOI:** 10.1186/1471-2407-12-259

**Published:** 2012-06-19

**Authors:** Daniel Weiss, Mario Koopmann, Türker Basel, Claudia Rudack

**Affiliations:** 1 Department of Otorhinolaryngology, Head and Neck Surgery, University Hospital Münster, Kardinal-von-Galen Ring 10, 48149, Münster, Germany

**Keywords:** Head and neck squamous cell carcinoma, *Cyclin A1*, Promoter methylation, Human Papillomavirus 16.

## Abstract

**Background:**

Promoter methylation of the tumor suppressor gene *Cyclin A1* could be associated with Human Papillomavirus 16 (HPV16) induced Head and Neck Squamous Cell Carcinoma (HNSCC) and Cervical Carcinoma. There is disagreement about the impact of this epigenetic event on protein expression of *Cyclin A1* in malignant and non-malignant tissue and there hardly exists any information about possible relationships between *Cyclin A1* expression and clinicopathological characteristics in HNSCC.

**Methods:**

We analyzed protein expression of *Cyclin A1* in 81 HNSCC and 74 benign tonsils by immunohistochemistry and correlated it to *Cyclin A1* methylation status, presence of HPV16 infection and other clinicopathological characteristics.

**Results:**

Overexpression of *Cyclin A1* was more present in HNSCC than in tonsils (p < 0.001). In both entities, HNSCC and benign tonsils, expression of *Cyclin A1* significantly correlated with the expression of Cyclin-dependent kinase-inhibitor *p16* (p = 0.000672 and 0.00495). In tonsils, expression of *Cyclin A1* was inversely proportional to age (p = 0.00000396), and further correlated with expression of tumor suppressor gene *p53* (p = 0.000228). In HNSCC *Cyclin A1* expression was associated with the presence of HPV16 DNA (p = 0.0014) and a lower recurrence rate in univariate and multivariate analysis (p = 0.002 and 0.013). Neither in HNSCC nor in tonsils *Cyclin A1* expression correlated with promoter methylation.

**Conclusions:**

*Cyclin A1* is an important cell cycle regulator with age-related increased expression in tonsils of children. HPV16 induces overexpression of *Cyclin A1* in HNSCC despite promoter methylation. Overexpression of *Cyclin A1* predicts a lower recurrence rate in HNSCC independently of HPV16.

## Background

In all types of tumors the cell cycle is an important target for both tumor progression and anti-tumor therapy. Activators and inhibitors are regulating the cell cycle through Cyclin-dependent kinase (CDK) complexes that catalyze the ordered transition from one phase of the cell cycle to the next [1]. The two known gene families of CDK inhibitors are the *cip*/*kip* and the *INK4a*/*ARF* family. The *cip/kip* family includes the genes *p21p27* and *p57*, which modulate the activity of, inter alia, the *Cyclin A*-CDK complex [2].

There are two A-type Cyclins, *Cyclin A1* and *Cyclin A2*. Both factors associate with CDK2, whereby *Cyclin A2* is essential for DNA replication and proliferation in somatic cells and *Cyclin A1* is highly expressed in testis and expressed at low levels in most other tissues [3,4]. *Cyclin A1* could bind to important cell cycle regulators: the *Rb* family of proteins, the transcription factor *E2F-1*, and the *p21* family of proteins [4]. *Cyclin A1* is required for S phase and passage through G_2_[5,6]. The disruption of the cell cycle through HPV by its oncogenes E6 and E7 is sufficiently known. In this connection, the most studied - and probably also the most important – cell cycle targets are *pRb* and *p53*[7]. However, HPV oncogenes can also abuse other important cell cycle regulators, like *Cyclin A*. Zerfass et al. could demonstrate that HPV16-E7 can transform rodent fibroblasts through induction of *Cyclin A* and *E*[8]. The transcription factor *E2F* forms a complex with p107, *Cyclin A*, and the CDK2 kinase (*E2Fcyclin A* complex) during S phase. HPV16-E7 associates very efficiently with the *E2FCyclin A* complex which is crucial for its transforming activity [4,9]. Malanchi et al. could demonstrate that not only HPV E7 but also E6 is able to promote G_1_/S transition through increasing the activity of the *Cyclin A*/Cyclin-dependent kinase 2 complex, which is involved in *pRb* phosphorylation. This leads to an accumulation of proteins that are negatively regulated by *pRb*, such as *p16*^*IKN4a*^*CDC2E2F-1* and *Cyclin A*[10,11].

Besides alcohol and nicotine abuse infection with high-risk HPV is one of the most important risk factors in the development of Head and Neck Squamous Cell Carcinoma (HNSCC) [12-14]. Methylation of *Cyclin A1* could be related to the presence of HPV16 DNA in Head and Neck Squamous Cell Carcinoma (HNSCC) and Cervical Neoplasia [15-17]. In cervical cancer *Cyclin A1* methylation could be associated with decreased protein expression and the integrated form of HPV. In contrast, we also found a higher frequency of *Cyclin A1* promoter methylation in HNSCC, especially in HPV16 positive tumors, but could not attribute it to reduced protein expression at first sight. We thought it was partly due to the low sample size.

Santopietro et al. could identify protein expression of *Cyclin A* as an independent predictor of high-risk HPV and high-risk HPV associated high-grade lesions in cervical cancer [18]. In renal, ovarian, and lung carcinoma cells *Cyclin A1* could be identified as a downstream target of *p53* that mediates apoptosis, G_2_/M arrest, and mitotic catastrophe if up-regulated [19,20]. An inverse correlation between *Cyclin A1* methylation and *p53* mutation in HNSCC could be shown by Tokumaru et al. [21]. Both research groups did their analysis without taking into account the HPV-status of cells. In our HNSCC collective we found a *p53*-independent influence of HPV16 on promoter methylation of *Cyclin A1*[15].

In this study we wanted to establish the impact of *Cyclin A1* expression on clinicopathological parameters in HNSCC. By including a larger sample size we intended to re-evaluate the relationship between HPV16 and *Cyclin A1*.

## Results

### Immunohistochemical expression of *Cyclin A1* in HNSCC and controls

**Figure 1:**
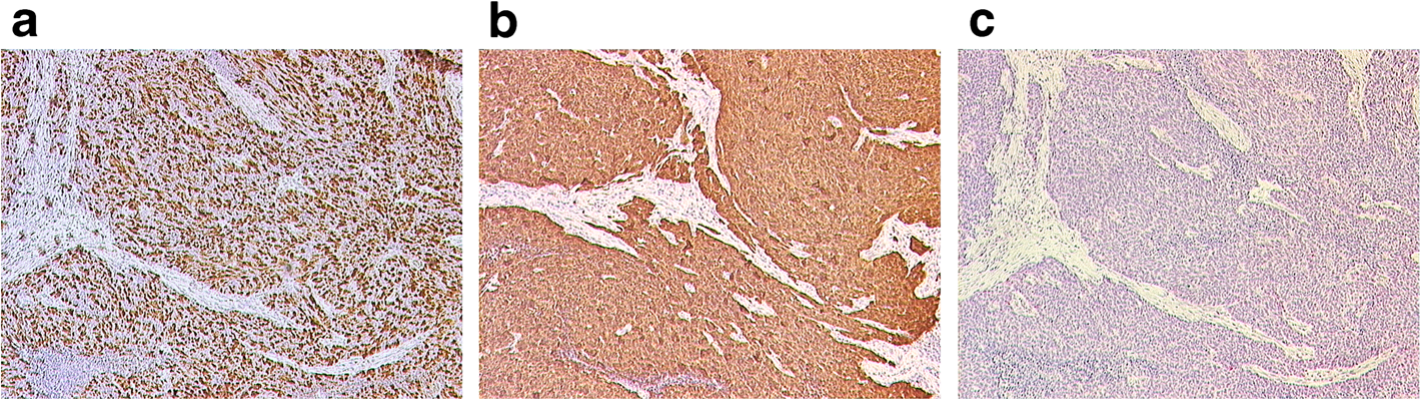
**Immunohistochemical staining of*Cyclin A1*(a),*p16*(b) and*p53*(c) in HNSCC.** Tumor of a 77 year old man; primary site: tonsil; HPV16 positive; *Cyclin A1* overexpression (category 8); *P16* overexpression (category 9); *P53* negative (category 1); original magnification: x50

**Figure 2:**
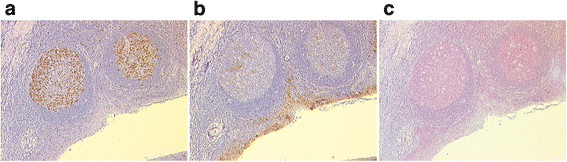
**Immunohistochemical staining of*Cyclin A1*(a),*p16*(b) and*p53*(c) in a benign tonsil.** Tonsil of a 41 year old man; HPV16 negative; *Cyclin A1* overexpression (category 4); *P16* category 2; *P53* category 2; original magnification: x50

In a first step we determined *Cyclin A1* expression in HNSCC (Figure 1a) and controls (benign tonsils, Figure 2a) through immunohistochemistry by three independent pathologists and categorized it into eleven different staining intensities (Table 1). On the basis of the expression results in tonsils and due to lack of reliable data in literature we set the cut-off for considering a *Cyclin A1* overexpression at ≥ 20% positive cells. Dependent (Chi-square test) or independent (Rank Sum Test) of a cut-off, the expression of *Cyclin A1* was much more intense in HNSCC compared to tonsils (p < 0.001). Due to disease-related epidemiology controls were much younger than patients with HNSCC (p < 0.001).

**Table 1:** **Results of HPV16 RT-PCR, immunohistochemistry of*p16*,*Cyclin A1*, and*p53*and*p53*mutation analysis**

		HNSCC (N = 81)	Controls/Tonsils (N = 74)	P
Age (mean ± sd)		62.78 ± 11.24	27.19 ± 19.26	<0.001
Sex	Male	53	40	0.200
	Female	28	34	
*Cyclin A1* overexpression (abs./%)	Total	24/29.6	4/5.4	<0.001
	Category 0 (negative)	0/0.0	2/2.7	
	Category 1 (few positive cells)	4/4.9	9/12.2	
	Category 2 (< 10%)	25/30.9	34/45.9	
	Category 3 (10-20%)	28/34.6	25/33.8	
	Category 4 (20-30%)	14/17.3	4/5.4	
	Category 5 (30-40%)	5/6.2	0/0.0	
	Category 6 (40-50%)	3/3.7	0/0.0	
	Category 7 (50-60%)	1/1.2	0/0.0	
	Category 8 (60-70%)	1/1.2	0/0.0	
	Category 9 (70-80%)	0/0.0	0/0.0	
	Category 10 (80-90%)	0/0.0	0/0.0	
	Category 11 (90-100%)	0/0.0	0/0.0	
HPV16 positive (abs./%)	Total	37/45.7	0/0.0	
	*P16* positive	29 out of 37/78.4		
	*Cyclin A1* positive	18 out of 37/48.6		
HPV16 negative (abs./%)	Total	44/54.3	74/100.0	
	*P16* positive	4 out of 44/9.1	0 out of 74/0.0	
	*Cyclin A1* positive	6 out of 44/13.6	4 out of 74/5.4	
*P16* overexpression (abs./%)	Total	33 out of 79/41.8	0/0.0	
	HPV16 negative	4 out of 33/12.1		
*P53* overexpression (abs./%)	Total	33 out of 80/41.3	0/0.0	
	HPV16 positive	4 out of 33/12.1		
*P53* mutation (abs./%)	Total	19 out of 41/46.3	0 out of 30/0.0	
	HPV16 positive	4 out of 19/21.1		
	*P53* positive	9 out of 19/47.4		

Cut off for overexpression in immunohistochemistry: *CDKN2A*/*p16* positive: ≥20% (category ≥4); *Cyclin A1* positive: ≥20% (category ≥4); *p53* positive: ≥10% (category ≥3).

### Prevalence of HPV16 DNA, overexpression of *p16* or *p53,* and *p53* mutation in HNSCC and controls

In HNSCC we found 37 (46%) HPV16 positive samples in Real-Time PCR (Table 1). Of those 37 HPV16 positive tumors, seven showed no overexpression of *p16*. Real-Time PCR revealed 44 HPV16 negative samples, whereby four samples showed an overexpression of *p16*. These samples were further analyzed for other HPV types by in-situ hybridization but remained HPV negative. When looking at the results broken down by primary site, tumors of the oropharynx (tonsil and base of the tongue) showed a substantially higher frequency of HPV16 and *p16* overexpression compared to non-oropharyngeal site (p < 0.001 and p = 0.008, Table 2). On the other hand tumors originating from the anterior two-thirds of the tongue showed markedly lower frequency of HPV16 (p = 0.035). Overexpression and mutation of *p53* could be detected in 33 of 80 and 19 of 41 tumor samples. Interestingly, four HPV16 positive tumors showed *p53* mutation but no *p53* overexpression and four HPV16 negative tumors showed overexpression of *p53* without *p53* mutation. Only about half of all tumors with *p53* mutation demonstrated overexpression of the associated protein in immunohistochemistry.

**Table 2:** **Prevalence of HPV16 positivity in Real-Time PCR and overexpression of*p16*in immunohistochemistry with respect to primary site**

Primary site	N	HPV16 positive RT-PCR			*P16* overexpression		
		abs.	%	P	abs.	%	P
Tonsil	33	19	57.6	0.112	15/32*	46.9	0.492
Base of the tongue	28	17	60.7	0.062	15/27*	55.6	0.094
Oropharynx (Tonsil and base of the tongue)	61	36	50.0	< 0.001	30/59*	50.8	0.008
Anterior two-thirds of the tongue	9	1	11.1	0.035	2	22.2	0.291
Floor of the mouth	2	0	0.0	0.498	0	0.0	0.507
Hypopharynx	1	0	0.0	1.000	0	0.0	1.000
Soft palate	2	0	0.0	0.498	0	0.0	0.507
Supraglottic	3	0	0.0	0.246	1	33.3	1.000
Unknown (CUP)	3	0	0.0	0.246	0	0.0	0.261

*P16 immunohistochemistry was done in 79 of 81 cases.

In tonsils we could not find any positive samples regarding HPV16 Real-Time PCR, overexpression of *p16* and overexpression of *p53*. *P53* mutation analysis revealed only wild-type samples.

### Correlation of *Cyclin A1* expression with clinicopathological parameters in controls

In benign tonsils the expression of *Cyclin A1* was inversely proportional to age (p = 0.00000396) (Table 3). Tissue sections from patients with daily nicotine consumption showed significantly lower expression rates than those of patients without nicotine abuse (p = 0.012). The expression of *Cyclin A1* showed weak but significant correlation with the protein expression of *p16* (p = 0.00495) and *p53* (p = 0.000228).

**Table 3:** **Correlation of*Cyclin A1*expression in immunohistochemistry with clinicopathological characteristics in controls (tonsils)**

Clinicopathological parameter	N	Performed Statistic	Subgroups	P
Age [y]	74	Spearman Rank Order	σ = −0.512	0.000004
Sex	74	Mann–Whitney Rank Sum	male vs. female	0.899
Nicotine abuse	74	Mann–Whitney Rank Sum	nicotine vs. no nicotine	0.012
Alcohol abuse	74	Mann–Whitney Rank Sum	alcohol vs. no alcohol	0.160
*P16* Expression	74	Spearman Rank Order	σ = 0.336	0.005
*P53* Expression	74	Spearman Rank Order	σ = 0.419	0.0002
*Cyclin A1* Methylation	12	Mann–Whitney Rank Sum	methylated vs. non-methylated	0.386

N: number of available samples; Age in years [y].

### Correlation of *Cyclin A1* expression with clinicopathological parameters in HNSCC

In HNSCC expression of *Cyclin A1* significantly correlated with HPV16-status (p = 0.001), with higher expression rates in HPV16 positive samples, but not with the copy number of E6 and E7 DNA (Table 4). Like in tonsils, the comparison of expression of *Cyclin A1* and *p16* revealed a relevant positive correlation between both variables (p = 0.000672). We could not find any correlation between the expression of *Cyclin A1* and the expression or mutation of *p53*. If correlated with other clinicopathological parameters, *Cyclin A1* expression showed no significant correlation with age, sex, primary site, tumor size, metastatic spread, Grading, UICC stage or alcohol and nicotine abuse (Table 4). As demonstrated earlier, *Cyclin A1* promoter methylation had no impact on *Cyclin A1* protein expression (p = 0.882) [15]. However, samples of HNSCC patients without locoregional recurrence showed significantly higher *Cyclin A1* expression than those of patients with locoregional recurrence (p = 0.002). This positive impact on the progression of the disease had no influence on survival, since we could not find improved progression-free or overall survival in patients with overexpression of *Cyclin A1* in associated samples. As expected, patients with HPV16 positive tumors had improved progression-free and improved overall survival (p = 0.017 and p = 0.018) (data not shown). We could also detect significantly more HPV16 positive samples in the group of patients without locoregional recurrence (p = 0.023). In Cox proportional-hazards regression overexpression of *Cyclin A1* represented an independent prognostic risk factor for lower recurrence rate (p = 0.013).

**Table 4:** **Correlation of*Cyclin A1*expression in immunohistochemistry with clinicopathological characteristics in HNSCC**

Clinicopathological parameter	N	Performed Statistic	Subgroups		P
Age [y]	81	Spearman Rank Order	σ = −0.070		0.532
Sex	81	Mann–Whitney Rank Sum	male vs. female		0.964
Primary site					
Tonsil	33	Fisher exact	*CCNA1* <4 vs. ≥4		0.623
Base of the tongue	28	Fisher exact	*CCNA1* <4 vs. ≥4		0.447
Anterior two-thirds of the tongue	9	Fisher exact	*CCNA1* <4 vs. ≥4		0.268
Floor of the mouth	2	Fisher exact	*CCNA1* <4 vs. ≥4		1.000
Hypopharynx	1	Fisher exact	*CCNA1* <4 vs. ≥4		1.000
Soft palate	2	Fisher exact	*CCNA1* <4 vs. ≥4		1.000
Supraglottic	3	Fisher exact	*CCNA1* <4 vs. ≥4		0.551
Unknown (CUP)	3	Fisher exact	*CCNA1* <4 vs. ≥4		0.208
T	72	Fisher exact	T_1/2_ vs. T_3/4_		0.591
N	73	Fisher exact	N_0/1_ vs. N_2/3_		0.312
M	75	Fisher exact	M_0_ vs. M_1_		0.094
G	62	Fisher exact	G_1/2_ vs. G_3/4_		0.404
UICC stage	71	Fisher exact	stage_1/2_ vs. stage_3/4_		1.000
Recurrence rate	64	Mann–Whitney Rank Sum	Recurrence vs. no recurrence	univariate	0.002
				multivariate	0.013
Secondary tumor	78	Mann–Whitney Rank Sum	sec. tumor vs no sec. tumor		0.516
Nicotine abuse	79	Mann–Whitney Rank Sum	nicotine vs. no nicotine		0.358
Alcohol abuse	79	Mann–Whitney Rank Sum	alcohol vs. no alcohol		0.743
Progression free survival	81	Kaplan-Meier	*CCNA1* <4 vs. ≥4		0.363
Overall survival	81	Kaplan-Meier	*CCNA1* <4 vs. ≥4		0.354
HPV16 DNA status	81	Mann–Whitney Rank Sum	HPV16+ vs. HPV16-		0.001
E6 quantitative	37	Spearman Rank Order	σ = 0.229		0.172
E7 quantitative	37	Spearman Rank Order	σ = 0.250		0.134
*P16* Expression	79	Spearman Rank Order	σ = 0.376		0.0007
*P53* Expression	80	Spearman Rank Order	σ = −0.086		0.448
*P53* Mutation	41	Mann–Whitney Rank Sum	*P53* mutated vs. wild-type		0.920
*Cyclin A1* Methylation	44	Mann–Whitney Rank Sum	methylated vs. non-methylated		0.882

N: number of available samples; CUP: Carcinoma of unknown primary; Age in years [y]; stage: UICC-stage; T: Tumor size: T1 (< 2 cm), T2 (2-4 cm), T3 (> 4 cm), T4 (Tumor infiltrates adjacent structures); N: status of regional lymph node metastasis: N0 (no metastasis), N1 (single ipsilateral lymph node metastasis, size < 3 cm), N2a (single ipsilateral lymph node metastasis, size 3-6 cm), N2b (multiple ipsilateral lymph node metastases, size 3-6 cm), N2c (multiple ipsi- and contralateral lymph node metastases, size 3-6 cm), N3 (lymph node metastasis, size > 6 cm); E6/E7 quantitative: results of quantitative HPV16 Real-Time PCR; *CCNA1*: *Cyclin A1*; *CCNA1* <4/≥4: immunohistochemical expression of *Cyclin A1* less than 20% or at least 20%.

## Discussion

We were the first to describe a connection between HPV16 and *Cyclin A1* promoter methylation in HNSCC [15]. In contrary to other reports, we could show in this context that the promoter methylation of *Cyclin A1* does not lead to a measurable impact on its protein expression and that the relationship between HPV16 and *Cyclin A1* methylation is independent of the expression or mutation of *p53*[19-21]. This time, we wanted to solve the jigsaw puzzle of HPV16 and *Cyclin A1* a little more by analyzing the protein expression of *Cyclin A1* in HPV16 positive and HPV16 negative HNSCC as well as in benign tonsils in a greater collective. We were further interested in detecting possible relationships between protein expression of *Cyclin A1* and clinicopathological characteristics in malignant and non-malignant oropharyngeal tissue. The results obtained by these investigations showed a strong correlation between *Cyclin A1* protein expression and HPV16 in HNSCC. To our great surprise, however, the expression of *Cyclin A1* depended more on the protein expression of *p16* than on the copy number of E6 and E7. It is assumed that *Cyclin A1* is induced by HPV16-E7 [4,8,9]. But, it could be proven that also the E6 oncogene of high and low risk HPV types is able to generate elevated *Cyclin A1* expression through phosphorylation of *pRb*. Santopietro et al. could identify protein expression of *Cyclin A* as independent predictor of high-risk HPV and high-risk HPV associated high-grade lesions in cervical cancer [18]. In cervical cancer no correlation was found between the quantity of HPV (determined by the proportion of HPV L1 and histone acetyltransferase – a human housekeeping gene) and *Cyclin A1* promoter methylation [17]. Yet, the physical state of HPV did affect methylation of *Cyclin A1*; the integrated form of HPV had a significantly higher impact on *Cyclin A1* methylation than the episomal form. We did not differentiate between integrated and episomal form of HPV in our HNSCC collective. It remains unclear if HPV16 induces promoter methylation or overexpression of *Cyclin A1* or both. Sartor and colleagues could determine a higher methylation frequency and a lower protein expression of *Cyclin A1* in HPV positive HNSCC than in HPV negative HNSCC [22]. They compared the methylation patterns and expression differences of various genes in one HPV16 positive cell line, two HPV negative cell lines and CaSki cells. What if HPV16 causes both, *Cyclin A1* promoter methylation and *Cyclin A1* protein overexpression? This would enable the virus to down regulate the tumor suppressive properties of *Cyclin A1* on the one hand and to have nevertheless the possibility of promoting cell cycle progression through *Cyclin A1* on the other hand. This exciting theory needs further clarification. Continuing experiments are already planned by our study group, particularly also because our results regarding *Cyclin A1* methylation and *p53* mutation are still limited by small sample size. Regardless of HPV, we could identify *Cyclin A1* as an independent risk factor of a lower recurrence rate in HNSCC. Nevertheless, overexpression of *Cyclin A1* did not have any impact on survival. Methylation of *Cyclin A1*, along with *DCC* and *CDKN2A*, has been found to predict longer disease-free survival in HNSCC [23]. In our collective, patients with *Cyclin A1* promoter methylation in their corresponding tumor samples tended to have improved progression-free and overall survival but without statistical significance (data not shown). Interestingly, *Cyclin A1* showed an age-related expression in tonsils. Younger patients, especially children under the age of 18 had markedly higher expression rates in corresponding tissue sections than older patients. We guess, that the increased expression of *Cyclin A1* in tonsils of little children reflects the enhanced immunological activity taking place in tonsils of patients in this age. This hypothesis is confirmed by the following facts: first, the expression of *Cyclin A1* was almost invariably found inside the centre of the lymphoid follicles of tonsils and, secondly, that the expression correlated with those of other markers for a heightened cell division rate, such as *p16* and *p53*. The elevated expression of *Cyclin A1* in tonsils of non-smokers could be due to the diminished or absent nicotine consumption in children. Because of epidemiological reasons controls were much younger than patients with HNSCC in our study. But, if we would redo the analysis of *Cyclin A1* protein expression with an age-matched control group, the statistical difference between both groups would become even larger.

## Conclusions

Taken together, the findings obtained in our study clearly demonstrate a linkage between HPV16 and *Cyclin A1* in HNSCC in one way or another. If the enhanced methylation frequency of *Cyclin A1* found in HNSCC and Cervical Cancer is directly caused by the HP-virus and the overexpression of *Cyclin A1* is only an indirect consequence of the altered cell cycle needs to be proven in further experiments. Irrespective of the relationship with HPV16 shown herein, *Cyclin A1* expression predicts recurrence rate in HNSCC and should therefore be considered as a possible tumor marker.

## Methods

### Patients and controls

We screened fresh-frozen tissue sections and formalin-fixed, paraffin-embedded tissue blocks of altogether 81 HNSCC from patients diagnosed and treated at the Department of Otorhinolaryngology, Head and Neck Surgery of the University Hospital of Münster, Germany. Patients’ age ranged from 30 to 89 years (mean: 62.78, standard deviation: 11.24 years). Fifty-three males and 28 females were included in the study (Table 1). Histological diagnose was based on examination of hematoxylin and eosin-stained tissue sections by at least two independent pathologists according to the World Health Organization Classification of Tumors [24]. The clinical data was abstracted from the patient’s medical chart. Preoperative evaluation included an endoscopy of the entire pharynx, larynx, trachea, bronchi and esophagus, a CT-scan of the head and neck, of the thorax and abdomen. Patients with lymph node metastasis but unknown primary (CUP) were referred to a F-18-fluordeoxyglucose positron emission tomography. In case of undetectable primary tumor these patients additionally received bilateral tonsillectomy and “blind” biopsies of the tongue base and epipharynx. All HNSCC were screened for HPV16 DNA in Real-Time PCR and expression of *CDKN2A*/*p16Cyclin A1*/*CCNA1*, and *p53* in immunohistochemistry. Analysis of *Cyclin A1* promoter methylation and *p53* mutation was done in 44 and 41 of these cases, respectively. The small sample sizes in both analyses are due to DNA fragmentation in Formalin-fixed paraffin-embedded tissue, which limits the amount of usable results.

Fresh frozen tissue sections from tonsils of 74 patients, which underwent tonsillectomy because of chronic tonsillitis or hyperplasia, were taken as controls (age: 27.19 ± 19.26 years; 34 females; 40 males) (Table 1). The study protocol was reviewed and approved by the ethics committee of the University of Münster, Germany, and informed consent was obtained from all patients.

### Quantitative HPV16 E6/E7 Real-Time PCR and HPV in-situ hybridization

Detection of HPV16 through Real-Time PCR and immunohistochemistry for *CDKN2A*/*p16* was done as described previously [25]. Briefly, tissue sections (20-μm) were cut and deparaffinized, and DNA was extracted using a commercial kit (QIAamp DNA Mini Kit). Real-time PCR was performed using ABI Prism 7900HT Sequence Detection System and the TaqMan Genotyping PCR Master Mix (Applied Biosystems) (Table 5). Beta-globin, a human housekeeping gene was used as an indicator for successful extraction of an equivalent length of target viral DNA. Serial dilutions of DNA isolated from cervical carcinoma cell line CaSki (ATCC-CRL-1550, 500 integrated HPV16 copies) were run in parallel as controls.

**Table 5:** Primers and probes used for HPV16-Real-Time PCR

Gene		Sequence (5’ → 3’)
HPV16 E6	Primer1, forward	CTGCAATGTTTCAGGACCCA
	Primer2, reverse	TCATGTATAGTTGTTTGCAGCTCTGT
	Probe	FAM-AGGAGCGACCCAGAAAGTTACCACAGTT-TAMRA
HPV16 E7	Primer1, forward	AAGTGTGACTCTACGCTTCGGTT
	Primer2, reverse	GCCCATTAACAGGTCTTCCAAA
	Probe	FAM-TGCGTACAAAGCACACACGTAGACATTCGTA-TAMRA
HBB	Primer1, forward	GTGAAGGCTCATGGCAAGAAAG
	Primer2, reverse	CAGCTCACTCAGTGTGGCAAAG
	Probe	FAM-ATGGCCTGGCTCACCTGGACAACC-TAMRA

PCR: Polymerase chain reaction; HPV16: Human Papillomavirus 16; E6, E7: Oncogenes of HPV16; HBB: Human beta-globin.

Tumors which were negative in the HPV16 Real-Time PCR but showed high *p16* expression were analyzed for presence of other HPV types by in-situ hybridization using the DNP-labelled genomic HPV probe sets INFORM HPV II Family 6 (HPV types 6, 11, 40, 42, 43, 44, 54, 61, 70, 72, 81) and HPV III Family 16 (HPV types 16, 18, 31, 33, 35, 39, 45, 51, 52, 56, 58, 59, 66, 68, 73, 82) (both from Ventana, Mannheim, Germany) and the automated Benchmark system (Ventana) according to the instructions of the provider. Briefly, 4 μm sections were deparaffinized, pre-treated with Protease 3, hybridized with both probe cocktails and stringently washed. Bound probes were subsequently detected by sequential incubation with anti-DNP antibodies, anti-Ig/Biotin, streptavidin bound alkaline phosphatase and BCIP. Previously analysed cervical biopsies were included as positive controls for both probe sets.

### Quantitation of HPV16 E6 and E7 DNA using Real-Time PCR

For Real-Time DNA Quantitation we generated two standard curves, one for determination of absolute DNA amount and one for calculation of E6 and E7 DNA amount [26]. The standard curve for absolute DNA amount was generated by amplification of β-globin using known amounts of genomic DNA from a healthy human individual (200.0 ng, 20.0 ng, 2.0 ng, and 0.2 ng; Promega, Madison, WI). The second standard curve for E6 and E7 DNA was generated by using known amounts of E6 or E7 PCR products obtained from CaSki cell DNA (1x10^8^ copies, 1x10^6^ copies, 1x10^4^ copies, and 1x10^2^ copies). The copy number of E6 or E7 DNA of tumor samples was determined by linear extrapolation of the E6 or E7 CT values of tumor samples, using the equation of the line obtained from the absolute E6 or E7 standard curve, and dividing these values by the relative amounts of β-globin.

### Immunohistochemical analysis of *Cyclin A1*, *CDKN2A/p16* and *p53*

For immunohistochemical analysis of *Cyclin A1CDKN2A/p16* and *p53* protein expression four micrometer-thick sections sliced from paraffin-embedded HNSCC specimens were deparaffinized by Xylene and subjected to antigen retrieval by microwaving in 10 mmol/L of sodium citrate for 30 minutes. The sections were incubated with a monoclonal mouse anti-human *Cyclin A1* antibody (6E6, Novocastra Laboratory, Newcastle United Kingdom) and with a monoclonal mouse anti-human *p53* antibody (DO-7, DakoCytomation, Glostrup, Denmark) for 60 min and 25 min, respectively, and stained by DAKO LSAB™+/HRP kit (No. K 0679, Dako, Glostrup, Denmark). For immunohistochemistry of *p16* (the protein encoded by *CDKN2A*) the CINtec® Histology Kit (mtm laboratories, Heidelberg, Germany) was used. For verification of specifity of staining an extra tissue section was stained with a monoclonal mouse anti-Rat oxytocin-related neurophysin antibody, as a negative control in each case. A HPV16-positive tumor with high *p16* expression was used as a positive control. The results of immunohistochemistry were based on examination of tissue sections by at least three independent pathologists (L.B., T.B. and G.K.) without knowledge of other clinicopathological data. Hematoxylin and eosin staining was performed for reference. The specimens were viewed with a Leica DMLB microscope. Strong nuclear staining was considered positive for *Cyclin A1* and *p53* expression. *P16* expression was scored as positive if strong and diffuse nuclear and cytoplasmic staining was present (Figure 1 and 2). Staining intensities were measured as the percentage of positively stained cells or nuclei in ten randomly selected fields (minimum of 1000 cells, 100x magnification). In the event of divergent results from one investigator or between investigators, the case was discussed again between the parties to find a consensus conclusion. In case of persistent differences between them, the mean of the three opinions was considered. The staining intensities for all three proteins were scored from 0–11 and allocated as follows: 0: negative specimens, 1: few positive cells or nuclei, 2: less than 10% positive cells or nuclei, 3: 10-20% positive cells or nuclei, 4: 20-30% positive cells or nuclei, 5: 30-40% positive cells or nuclei, 6: 40-50% positive cells or nuclei, 7: 50-60% positive cells or nuclei, 8: 60-70% positive cells or nuclei, 9: 70-80% positive cells or nuclei, 10: 80-90% positive cells or nuclei, 11: 90-100% positive cells or nuclei. The cut-off for considering an overexpression was deduced from the results of those achieved for the benign tonsils and those of literature [27-29]. They were in detail for the specific proteins: *CDKN2A*/*p16*: ≥ 20% (IHC category ≥ 4), *Cyclin A1*/*CCNA1*: ≥ 20% (IHC category ≥ 4), and *p53*: ≥ 10% (IHC category ≥ 3).

### Methylation specific Polymerase Chain Reaction

For methylation analysis of *Cyclin A1* in tumor and control tissue the Methyl-Profiler™ DNA Methylation PCR Array System (SABiosciences, Frederick, USA) was used. This system can detect DNA methylation of up to 96 genes simultaneously with pre-designed primers but without bisulfite conversion. DNA methylation-sensitive and methylation-dependent restriction enzymes are used to selectively digest unmethylated or methylated DNA, respectively. The remaining DNA after digestion is quantified by Real-Time PCR using primers that flank the region of interest. The relative concentration of differentially methylated DNA (specifically hypermethylated, intermediately methylated, and unmethylated DNA) are determined by comparing the amount in each digest with that of a mock digest. The PCR cycling conditions were as follows: 1 cycle at 95 °C for 10 minutes, 40 cycles including 15 seconds at 97 °C and 1 minute at 72 °C. The PCR product was marked with SYBR® Green. According to the manufactures protocol the cut-off level for significant promoter methylation should be defined by the user. A sample or tumor can be defined as significantly methylated, if the extent of hypermethylated DNA reaches 10 to 20 percent irrespective of the intermediately methylated fraction. However, this threshold is dependent on the extent of non-target cell contamination. The greater the extent of contamination, the higher the threshold must be set. We decided to set the cut-off level at 20% to detect only the truly methylated samples. Methylation analysis was done in 44 tumor and 12 control samples.

### Mutation analysis of *p53*

DNA was extracted from tumor tissue and tonsils as described above. Polymerase chain reaction (PCR) was used to amplify the domain regions and flanking splice sites of exons 2–9 of the *p53* gene, using primer sets and conditions described in Table 6. PCR was performed in a 25 μl volume containing 200 ng of genomic DNA; 1xPCR buffer (Invitrogen, Burlington, Canada); 5 nmol of each dCTP, dGTP, dTTP, and dATP; 1.5 mM MgCl2; 15pmol of each primer; and 1 U of Taq polymerase (Invitrogen). The PCR conditions were 165 sec at 96 °C, 45 sec at appropriate annealing temperature and 9 min at 72 °C. The PCR products were sequenced using the ABI Prism 3730 DNA Analyzer (Applied Biosystems, Foster City, CA, USA). *P53* mutational status was estimated in 41 HNSCC patients and 30 controls. Sequence chromatograms were compared with the *p53* reference sequence in the IARC *TP53* Mutation Database (www-*p53*.iarc.fr) and/or the Single Nucleotide Polymorphism (SNP) Database (http://www.ncbi.nlm.nih.gov/projects/SNP/).

**Table 6:** **Primers and probes used for*p53*mutation analysis**

Exon	Primer		Sequences (5’ → 3’)	Annealing Temperature (°C)
2-4	A1	forward	TCAGACACTGGCATGGTGTTG	64
		reverse	AGGGTGAAGAGGAATCCCAAAG	
	A2	forward	GTGAGTGGATCCATTGGAAG	64
		reverse	GTGAAAAGAGCAGTCAGAGG	
5/6	B	forward	TAGTGGGTTGCAGGAGGTGC	62
		reverse	GAGGCCCTTAGCCTCTGTAAGC	
7	C	forward	CTGCTTGCCACAGGTCTCC	62
		reverse	CGGGGATGTGATGAGAGGTGG	
8/9	D	forward	AAGGACAAGGGTGGTTGGGAG	62
		reverse	CAACCAGGAGCCATTGTCTTTG	

### Statistical analysis

The clinical and pathological data was analyzed by Fisher’s exact test for categorical variables and Mann–Whitney test for continuous variables. Possible correlation between two continuous variables was analyzed by Spearman rank order. Differences in survival probability were compared by Kaplan-Meier LogRank, whereby death from any cause was considered an event and data on patients who were alive at the last follow-up contact were censored. These analyses were performed using SigmaPlot software. Statistical significance was accepted at p < 0.05.

## Abbreviations

HNSCC: Head and neck squamous cell carcinoma; HPV: Human papilloma virus; CUP: Carcinoma of unknown primary; CDK: Cyclin-dependent kinase; RT-PCR: Real-Time PCR.

## Competing interests

The authors declare that they have no competing interests.

## Authors’ contributions

DW participated in the design of the study, acquisition of data, and interpretation of data and helped to draft the manuscript. MK participated in the design of the study and helped performing the statistical analysis. TB participated in methylation analysis and *p53* mutation analysis. CR conceived of the study, and participated in its design and coordination and helped to draft the manuscript. All authors read and approved the final manuscript.

## Pre-publication history

The pre-publication history for this paper can be accessed here:

http://www.biomedcentral.com/1471-2407/12/259/prepub
